# Knowledge and attitudes towards smoking cessation counselling: an Italian cross-sectional survey on tertiary care nursing staff

**DOI:** 10.7717/peerj.12213

**Published:** 2021-10-15

**Authors:** Laura Maniscalco, Salvatore Barretta, Giuseppe Pizzo, Domenica Matranga

**Affiliations:** 1 Department of Biomedicine, Neuroscience and Advanced Diagnostics, University of Palermo, Palermo, Italy; 2 Department of Health Promotion, Mother and Child Care, Internal Medicine and Medical Specialties, University of Palermo, Palermo, Italy; 3 Department of Surgical, Oncological and Oral Sciences, University of Palermo, Palermo, Italy

**Keywords:** Smoking cessation, Counselling, Education, Training, Tobacco-control, Quit smoking

## Abstract

**Background:**

One of the most effective smoking cessation strategies involves care and advice from nurses due to their role in the front line of treatment. Lack of education on smoking cessation counselling may be detrimental, and adequate smoking cessation training during healthcare studies is needed.

**Objectives:**

The study aimed to examine nurses’ attitudes, belief, and knowledge of smoking cessation counselling; knowledge of the health risks associated with smoking was also assessed.

**Design:**

A cross-sectional survey on 77 nurses from the nursing staff of Cardiology, Cardiac Intensive Care and Surgical Oncology Units of two tertiary hospitals.

**Methods:**

Cronbach’s alpha was calculated to assess the questionnaire’s internal consistency, and three composite indicators were computed to assess the three dimensions of the questionnaire (knowledge, attitude, belief). Furthermore, a stepwise linear regression model was used to predict the attitude to be engaged in smoking cessation counselling, related to demographic and behavioural variables, as well as knowledge and belief indicators. The analysis was stratified by Unit.

**Results:**

Nurses from three Units had a significantly different attitude score (2.55 ± 0.93 for Cardiology, 2.49 ± 0.72 for Cardiac Intensive Care and 2.09 ± 0.59 for Surgical Oncology Unit) (*P*-value = 0.0493). Analogously, knowledge of smoking cessation counselling was reported to be higher for Cardiac Intensive Care Unit nurses (3.19 ± 0.70) compared to Surgical Oncology nurses (2.73 ± 0.74) (*P*-value = 0.021). At the multivariable analysis, attitude towards smoking cessation counselling was significantly related to the nurse’s belief about counselling, for Cardiology staff (coeff = 0.74, 95% CI [0.32–1.16], *P*-value = 0.002) and for Surgical Oncology staff (coeff = 0.37, 95% CI [0.01–0.72], *P*-value = 0.042).

**Conclusions:**

Incorporation of smoking cessation interventions in nurses’ and nursing managers’ education could improve the nursing staff’s attitude, belief, and knowledge regarding smoking cessation counselling, which would lead to the inclusion of tobacco prevention and cessation as an integral part of patient care.

## Introduction

Smoking is one of the biggest concerns of public health, as it is responsible for more than eight million deaths per year worldwide (WHO, 2020). The Global Burden of Diseases report estimated that 25% of men and 5% of women smoke every day, equaling about one billion people in the world (Reitsma et al., 2017). The impact of smoking in 2012 on world healthcare costs was $422 billion for direct care and $1.436 billion for indirect care, related to the productivity loss due to illness or death, equal to 1.8% of the annual world Gross Domestic Product (GDP) (Goodchild, Nargis & d’Espaignet, 2018). In Europe, the proportion of daily smokers is 24%, varying from a minimum of 5% in Sweden, it is 16% in the United Kingdom, Netherlands, and Denmark and it reached a percentage higher than 30% in some European countries (*e.g*. 36% Bulgaria, 35% Greece, 33% Croatia, 33% France) (Directorate-General for Communication (European Commission), Directorate-General for Health and Food Safety (European Commission), TNS Opinion & Social, 2015). In Italy, the proportion of smokers is lower than the European average, but it is still high (18.4%). Furthermore, in 2016 the Italian National Institute of Health in 2016 estimated that the national healthcare expenditure for smoking-related diseases amounted to 26 billion euros (Ministero della Salute, 2020). A recent study conducted in Italy, Spain and Greece confirmed that smoking habits and other lifestyle indicators, such as weight change and exercise, are strictly associated with non-communicable diseases including cardiovascular diseases, cancer, chronic respiratory diseases (Maniscalco et al., 2020). Specifically, tobacco was associated with at least 25 diseases. It is estimated that 71% of lung diseases, 42% of chronic obstructive pulmonary diseases, 10% of cardiovascular diseases, 22% of all cancer deaths and 12% of all causes of death are smoking-related (WHO, 2012). Indeed, in Italy, more than 25% of smoking-related deaths affects subjects between 35 and 65 years. Among the major smoking-related diseases, in 2016 mortality and incidence of lung cancer has decreased among men, but it is the third cause of cancer deaths among women, after breast and colorectal cancer (Ministero della Salute, 2020). One of the priorities established by the WHO’s Agenda 2030 for health (WHO, 2020, 2004) is the reduction of demand and supply of tobacco. Emerging evidence shows that one of the most effective smoking cessation strategies involves health professional advice and care (Fiore et al., 2011; Schwartz, 1992), especially if it is provided by nurses on duty at the front line of treatment, as they spend a lot of time with their patients (Rice et al., 2017). Recent literature showed that smoking cessation counselling from health professionals is very effective to help patients quitting smoking (Fiore et al., 2011). A systematic review showed a significant increase in the rate of quitting after brief smoking counselling from health professionals compared to the usual care (Risk Ratio (RR) = 1.66, confidence interval (CI) [1.42–1.94]) (Stead et al., 2013). More recently, a Cochrane review of 44 RCTs of smoking cessation interventions delivered by nurses, using the same outcome as above, showed that the intervention significantly increased the probability of abstinence 6 months apart (RR 1.29, 95% CI [1.21–1.38]). However, the quality of evidence was judged from moderate to low (Rice et al., 2017).

The limited activity of smoking cessation counselling among health professionals can be due to have received an insufficient training during undergraduate and postgraduate studies (Shishani et al., 2013). Not with standing the importance of smoking cessation counselling, many nursing schools have not still integrated smoking cessation education into their curriculum. Studies conducted in the US and other countries like New Zealand, China, Japan, Korea, and the Philippines, showed that the time spent in nursing education programs to assess the contents, time and techniques to be used in tobacco cessation interventions was negligible (Petersen et al., 2017). As nursing students do not receive adequate theory or practical education regarding smoking cessation counselling, there is an urgent need to develop innovative and evidence-based tobacco-use cessation programs and integrate these into nursing curricula.

To the best of our knowledge, there are very few data about smoking cessation content in the Italian healthcare curricula for both bachelor and master’s degree courses in Nursing. La Torre et al. (2014) reported that 17% of healthcare professionals were not satisfied with their university training on smoking cessation. Other authors showed that the percentage of medical and healthcare students who received specific training on smoking cessation was between 9.4% (Armstrong et al., 2017) and 24% (D’Egidio et al., 2020). Furthermore, Grassi et al. (2012) found that Italian medical students did not receive adequate smoking cessation training during medical school.

This study aimed to examine nurses’ attitudes, belief, and knowledge of smoking cessation counselling in the Cardiology Unit (CU), Cardiac Intensive Care Unit (CICU) and Surgical Oncology Unit (SOU) of two tertiary Hospitals. Knowledge of the health risks associated with smoking was also assessed.

## Materials & methods

### The study population

A cross-sectional survey was conducted on the nursing staff of the CU, CICU and SOU of two tertiary hospitals, in the period February-April 2017. The study included 77 nurses (out of an entire staff of 86, 89.5% response rate), enrolled at two CUs (19 nurses), at two CICUs (27 nurses) and at two SOUs (31 nurses). Respondents were raised about the importance to fill out the whole questionnaire. It is calculated that 77 nurses are sufficient to estimate the proportion of nurses receiving specific training on smoking, as to guarantee a confidence level of 95% and an absolute error of 10%. We assumed that the proportion of nurses receiving specific training on smoking was equal to the proportion of 24% as estimated by D’Egidio et al. (2020) for healthcare students. Non-respondents (9/86) were excluded from the study. The survey technique consisted of a self-administered paper questionnaire.

### Ethics statement

The study has been performed in accordance with the principles set forth in the Helsinki Declaration. The study protocol was approved by the Institutional Review Board of Azienda Ospedaliera Paolo Giaccone of Palermo (approval number 3/2017). Written informed consent was obtained from all participants.

### The questionnaire

The questionnaire used to collect data from nurses was based on an earlier version developed by the European Union Working Group on Tobacco and Oral Health (Pizzo et al., 2010) and adapted to the characteristics of the nurses. The questionnaire was Italian-written and divided into three sections (See Supplementary Materials). The draft of this questionnaire was reviewed by three expert nurses within “Paolo Giaccone” University Hospital to check its completeness and its suitability to be used to assess knowledge and attitudes towards smoking cessation counselling of nurses.

In the first section, the nurses were asked to indicate socio-demographic information, namely gender, age, education and hospital unit to which they belong. Education was categorized into three responses (Nursing school according to the Italian law nr. 42/1999, nursing diploma, bachelor’s and master’s degree). In the second section, nurses chose from a list of common diseases for which, in their opinion, smoking could be indicated as a significant risk factor (question no.1). Furthermore, they were asked about their personal smoking habits (questions no. 2 and 3) and about their satisfaction with the training received during their studies about smoking-related harm to health (question 4). Finally, the third section was aimed at detecting the nurse’s knowledge (questions 5 and 6), the nurse’s attitude (question 7) and the nurse’s personal belief (question 8) about smoking cessation counselling.

In order to detect the respondent’s smoking habits, question 2 distinguished among current smoker, former smoker and non-smoker, while question 3 was addressed to smokers only and asked for open answers. The items proposed in questions no. 2–8 were measured on a 5-point Likert scale with the following anchors: 1, Absolutely not; 2, Not; 3, Quite; 4, Yes; and 5, Absolutely yes.

### Statistical methods

Categorical variables were expressed as counts and percentages, continuous variables as mean and standard deviation. Age was categorized into two classes, ≤40 *vs* >40. The statistical association between categorical variables was assessed through either the Chi-square test or the Fischer exact test, as appropriate. The mean scores of all questions answered by the three hospital units’ nurses were assessed through the one-way ANOVA. The normality assumption was assessed through the Shapiro–Wilk test and the homoscedasticity assumption through the Bartlett’s test. In case of statistical significance, post-hoc multiple comparisons have been made with Sidak’s multiplicity correction. Cronbach’s alpha was calculated to assess the questionnaire’s internal consistency. For each one of three questionnaire’s dimensions (knowledge, attitude, belief), composite indicators were obtained as the simple arithmetic mean of the scores reported to the single items (questions no. 5–6 for knowledge, question no. 7 for attitude and question no. 8 for belief). To compute composite indicators, items with negative direction were reversed to give a score of one to the maximum and five to the minimum. Finally, stepwise linear regression model was used to predict the attitude to smoking cessation counselling related to demographic and behavioral variables, in addition to knowledge and belief indicators. In the stepwise linear regression, the significance level was *P*-value = 0.10 for removal of a variable from the model and *P* = 0.05 for addition of a variable to the model. The hospital unit was considered as stratification variable for the stepwise linear regression analysis. Statistical analysis was performed using Stata IC/15.1 (Stata Corporation, College Station, TX, USA). A *P*-value < 0.05 was used as cut-off value for statistical significance.

## Results

The sample included 77 nurses of which 19 (25%) of CUs, 27 (35%) of CICUs and 31 (40%) of SOUs. Considering the staff’s distribution by hospital units, the surveyed nurses were homogeneous for gender and education. However, those working at the SOU were younger on average compared to the other Units (48.4% was less than forty years old, compared to 14.8% and 10.5% of CICU and CU, respectively). There was no statistically significant difference in the smoking habits of the interviewed nurses, with more than half reporting to be non-smokers and one quarter being smokers (Table 1).

**Table 1:** Descriptive statistics of the interviewed nurses by hospital unit.

Variables	CU^a^ (*n* = 19)	CICU^a^ (*n* = 27)	SOU^a^ (*n* = 31)	Total	*P*-value^b^
Gender					0.751
Male, *n* (%)	10 (52.6)	13 (48.2)	18 (58.1)	41 (53.3)	
Female, *n* (%)	9 (47.4)	14 (51.9)	13 (41.9)	36 (46.8)	
Age					0.003
≤40, *n* (%)	2 (10.5)	4 (14.8)	15 (48.4)	21 (27.3)	
>40, *n* (%)	17 (89.5)	23 (85.2)	16 (51.6)	56 (72.7)	
Education					0.124
Nursing school, *n* (%)	11 (57.9)	19 (70.4)	12 (38.7)	42 (54.6)	
Nursing diploma, *n* (%)	2 (10.5)	4 (14.8)	9 (29.0)	15 (19.5)	
Master’s and bachelor’s Degree, *n* (%)	6 (31.6)	4 (14.8)	10 (32.3)	20 (26.0)	
Smoking status					0.970
Not smoker, *n* (%)	10 (52.6)	15 (55.6)	18 (58.1)	43 (55.8)	
Smoker, *n* (%)	5 (26.3)	7 (25.9)	6 (19.4)	18 (23.4)	
Former smoker, *n* (%)	4 (21.1)	5 (18.5)	7 (22.6)	16 (20.8)	
Satisfaction with own education					0.122
No, *n* (%)	6 (31.6)	10 (37.0)	18 (58.1)	34 (44.2)	
Yes, *n* (%)	13 (68.4)	17 (63.0)	13 (41.9)	43 (55.8)	

**Notes:**

a CU, Cardiac Unit; CICU, Cardiac Intensive Care Unit; SOU, Surgical Oncology Unit.

b Chi-square test was used

Regarding their personal opinions about the association between some chronic diseases and smoking, there was a statistically significant difference for a few diseases. Cardiac and vascular diseases and gastric and duodenal ulcers were associated with smoking more by CU and CICU nurses than by SOU (*P*-value = 0.003 and *P*-value = 0.028, respectively, for two diseases). On the other hand, complications in pregnancy were considered in relation to smoking by SOU nurses (87%) and far less by other nurses (*P*-value = 0.001) (Table 2).

**Table 2:** Diseases associated with smoking habit in the opinion of the interviewed nurses, by hospital unit.

Variables	CU^a^ (*n* = 19)	CICU^a^ (*n* = 27)	SOU^a^ (*n* = 31)	*P*-value^b^
Cancer, *n* (%)	19 (100.0)	26 (96.3)	28 (90.3)	0.297
Respiratory diseases, *n* (%)	19 (100.0)	27 (100.0)	30 (96.77)	0.472
Vascular and cardiac diseases, *n* (%)	19 (100.0)	26 (96.3)	22 (70.97)	0.003
Infertility, *n* (%)	9 (47.37)	12 (44.44)	9 (29.03)	0.334
Impotence, *n* (%)	14 (73.68)	13 (48.15)	21 (67.74)	0.154
Complications in pregnancy, *n* (%)	8 (42.11)	13 (48.15)	27 (87.10)	0.001
Cataract, *n* (%)	4 (21.05)	3 (11.11)	1 (3.23)	0.132
Gastric and duodenal ulcer, *n* (%)	8 (42.11)	11 (40.74)	4 (12.90)	0.028

**Notes:**

a CU, Cardiac Unit; CICU, Cardiac Intensive Care Unit; SOU, Surgical Oncology Unit.

b Chi-square test was used.

The questionnaire’s internal consistency was good (Cronbach’s alpha = 0.8417). Regarding nurses’ knowledge about smoking cessation, nurses of three units were significantly different for answers to questions Q5a (*P*-value = 0.006), Q5c (*P*-value = 0.006) and Q6 (*P*-value = 0.014). Specifically, nurses of CU and CICU were more in agreement than nurses of SOU with the sentence Q5a “The inpatient ward is the ideal place to provide information on the health harm caused by cigarette smoking” (*P*-value = 0.019 and *P*-value = 0.023, respectively). Nurses of CU were more in agreement with the sentence Q5c “If the patient decides to quit smoking, the nurse must set a precise start date of smoking cessation” than nurses of CICU (*P*-value = 0.006) and SOU (*P*-value = 0.029). Nurses of CICU agreed significantly more than those of SOU with the sentence Q6 “The nurse must report the smoking status of each patient on the medical record (also in electronic format)” (*P*-value = 0.015). Looking at the composite indicator of Nurse’s Knowledge, the score obtained for CICU (3.19 ± 0.70) was higher than that one obtained for SOU (2.73 ± 0.74) (*P* = 0.0.045) (Table 3).

**Table 3:** Answers to questions to assess nurse’s knowledge, attitude and belief about counselling for smoking cessation.

Questions^b^	CU^a^ (*n* = 19)	CICU^a^ (*n* = 27)	SOU^a^ (*n* = 31)	*P*-value^c^	Post-hoc comparisons^d^
Q5a. The inpatient ward is the ideal place to provide information on the health harm caused by cigarette smoking	3.05 ± 1.18	2.96 ± 0.98	2.29 ± 0.69	0.006	CICU *vs* SOU(*P-*value = 0.019) CU *vs* SOU(*P*-value = 0.023)
Q5b. The nurse must always ask the smoker patient weather she/he wants to quit smoking	2.80 ± 1.27	2.88 ± 1.01	2.77 ± 0.80	0.902	
Q5c. If the patient decides to quit smoking, the nurse must set a precise start date of smoking cessation	3.00 ± 0.94	2.04 ± 1.20	2.22 ± 0.84	0.006	CICU *vs* CU(*P*-value = 0.006) SOU *vs* CU(*P*-value = 0.029)
Q5d. The nurse must always explain to the smoker patient the harmful effects of smoking on health	3.42 ± 1.22	3.78 ± 1.01	3.03 ± 1.25	0.058	
Q6. The nurse must report the smoking status of each patient on the medical record (also in electronic format)	3.26 ± 0.87	3.44 ± 1.09	2.68 ± 1.01	0.014	CICU *vs* CU(*P*-value = 0.015)
*Composite indicator of Nurse’s Knowledge*	3.21 ± 0.68	3.19 ± 0.70	2.73 ± 0.74	0.021	CICU *vs* CU(*P*-value = 0.045)
Q7a. The nurse must do counselling interventions to smoking cessation in the context of clinical activity	2.89 ± 0.94	3.11 ± 1.15	2.84 ± 0.86	0.561	
Q7b. It is difficult to explain the health benefits deriving from smoking cessation because it takes time away from nursing care (reversed item)	2.24 ± 1.29	2.78 ± 1.31	1.61 ± 1.08	0.002	CICU *vs* CU(*P*-value = 0.002)
Q7c. In general, the nurse has limited knowledge of the negative effects of smoking on health (reversed item)	2.57 ± 1.28	2.04 ± 1.26	1.85 ± 0.85	0.094	
Q7d. The nurse is not able to help the smoker patient to stop smoking (reversed item)	2.50 ± 1.56	2.04 ± 1.48	2.06 ± 1.14	0.463	
*Composite indicator of Nurse’s Attitude*	2.55 ± 0.93	2.49 ± 0.72	2.09 ± 0.59	0.049	
Q8a. The nurse who advises smoker patients to quit smoking makes them uncomfortable (reversed item)	2.11 ± 1.51	1.39 ± 1.17	1.53 ± 1.06	0.133	
Q8b. If the nurse advises the smoker patient to stop smoking, the patient considers him a moralist (reversed item)	1.91 ± 1.52	1.04 ± 1.39	2.06 ± 1.0	0.918	
Q8c. Patients appreciate the nurse who advises them to stop smoking	2.95 ± 1.22	2.40 ± 1.12	2.48 ± 0.93	0.212	
Q8d. The nurse who advises patients to stop smoking will cause many smokers to quit smoking	2.63 ± 1.01	2.07 ± 0.83	2.29 ± 0.82	0.111	
Q8e. The nurse who advises patients to stop smoking gives a good picture of him/herself	2.84 ± 1.12	2.37 ± 0.93	2.64 ± 0.98	0.287	
Q8f. The nurse who advises patients to stop smoking will help reduce the incidence of cardiovascular disease and other smoking-related diseases among smokers	3.79 ± 0.98	3.33 ± 0.96	2.87 ± 1.17	0.014	CU *vs* CU (*P*-value = 0.012)
*Composite indicator of Nurse’s Belief*	2.70 ± 0.85	2.27 ± 0.65	2.31 ± 0.57	0.074	

**Notes:**

a CU, Cardiac Unit; CICU, Cardiac Intensive Care Unit; SOU, Surgical Oncology Unit.

b Mean and (SD).

c Anova test was used.

d Only statistically significant Post-hoc comparisons were shown.

Regarding nurses’ attitude towards smoking cessation counselling, answers to question Q7b were significantly different among the three hospital units. In particular, the CICU staff was more in agreement than the SOU staff with the sentence “It is difficult to explain the health benefits deriving from smoking cessation because it takes time away from nursing care” (*P*-value = 0.002). The three units’ nurses were significantly different for the composite indicator of Nurse’s Attitude (2.55 ± 0.93 for CU, 2.49 ± 0.72 for CICU and 2.09 ± 0.59 for SOU) (*P*-value = 0.049) but none of the post-hoc paired comparisons were remarkable (Table 3).

With regards to nurses’ belief about smoking cessation counselling, answers to question Q8f were significantly different among the three hospital units (*P*-value = 0.014). Nurses of CU were more in agreement with the sentence “The nurse who advises patients to stop smoking will help reduce the incidence of cardiovascular disease and other smoking-related diseases among smokers” than nurses of SOU (*P*-value = 0.012). Looking at the composite indicator of Nurse’s Belief about smoking cessation counselling, the staff from three hospital units could be considered homogeneously levelled down ((2.17 ± 0.55 for CU, 1.81 ± 0.46 for CICU and 1.83 ± 0.76 for SOU) (*P*-value = 0.074)) (Table 3).

Results of multivariable analysis showed that attitude to smoking cessation counselling was positively related to belief for nurses working in CU (coeff = 0.74, 95% CI [0.32–1.16], *P*-value = 0.002) and SOU (coeff = 0.37, 95% CI [0.01–0.72], *P*-value = 0.042) while the association with knowledge was borderline significant (Table 4).

**Table 4:** Attitude to patient counselling for smoking cessation in relation to demographic and behavioral variables, knowledge and belief indicators, by hospital unit-multivariable analysis.

Covariates	CU^a^		CICU^a^		SOU^a^	
	Coefficient (95% CI)	*P*-value	Coefficient (95% CI)	*P*-value	Coefficient (95% CI)	*P*-value
Intercept	0.55 [−0.64 to 1.73]	0.344	1.53 [0.53–2.53]	0.004	0.50 [−0.38 to 1.38]	0.257
Belief	0.74 [0.32–1.16]	0.002	0.43 [0.00–0.85]	0.050	0.37 [0.01–0.72]	0.042
Knowledge					0.27 [0.00–0.54]	0.047

**Note:**

a CU, Cardiac Unit; CICU, Cardiac Intensive Care Unit; SOU, Surgical Oncology Unit.

## Discussion

Healthcare workers play an essential role in promoting smoking cessation and tobacco addiction treatment (Nilan et al., 2019; WHO, 2005). Smoking cessation has been established as one of the six ways to get healthy, together with regular physical exercise, adequate and good quality sleep, stress management and maintenance of relationships (Polak, Pojednic & Phillips, 2015). Previous works (Cerame et al., 2008, Matranga et al., 2020) demonstrated the need to equip the students destined to work in the field of healthcare with counselling skills for a more effective promotion of healthy lifestyles in the general population. As health experts and promoters, nurses are the most important health professionals to deliver smoking cessation counselling (Rice et al., 2017). According to the National Institute for Health and Clinical Excellence (NICE) Guidelines, nurses should ask patients about smoking and advise them of the dangers of exposure to active and passive smoke (Linden, 2019). Their strategic role is due to the excellent position to raise the issue with patients and signpost them to the appropriate local support services (Carlebach & Hamilton, 2009).

Our study showed that CU, CICU and SOU nurses showed considerable knowledge regarding the association between some chronic diseases and smoking. The majority of nurses in our study suggested the three most important chronic diseases associated with smoking are cancer, respiratory diseases, and cardiovascular diseases. This result is crucial as insufficient knowledge of smoking risks has been suggested as one of the most important barriers to nurses’ role as educators (Derksen et al., 2019). The higher knowledge of CICU nurses may be explained by the fact that these professionals treat patients who are more likely to change their lifestyle. Clark & Moss (2011) defined the period of abstinence offered by hospitalization for a medical illness as a “teachable moment”. Patients aware that their hospitalization at ICU was smoking-related were more likely to have quit smoking at the 6-month follow-up (Polmear et al., 2017). Therefore, the experience of ICU admission seems to influence the attitude of patients towards smoking cessation counselling. Analogously, smokers who experienced cardiovascular bypass surgery were more likely to quit (Rice et al., 1994). The Surgical Oncology nurses’ awareness in our sample is in line with current literature, where they seemed to be of significant support and could be considered as educators for smokers after cancer diagnosis (Cooley et al., 2008). Previous studies reported that most nurses believed that their role in smoking cessation interventions is fundamental and that they should advise patients to quit smoking (Lang, Waterworth & O’Brien, 2018; McCarty et al., 2001). Our study showed a large consensus among the nurses about the belief that the inpatient ward is the ideal place for providing information regarding the health harm caused by cigarette smoking, especially for CU and CICU nurses.

In our study, nurses found that it was difficult to explain the health benefits deriving from smoking cessation because it takes time away from nursing care. Similarly, Nagle, Schofield & Redman (1999) reported that the nurses believed they were too busy to teach their patients about stopping smoking, whereas other authors reported that nurses stated that giving smoking cessation advice was not part of their responsibility (Raupach et al., 2014; Svavarsdóttir & Hallgrímsdóttir, 2008). On the contrary, Lang, Waterworth & O’Brien (2018) found that both physicians and nurses considered smoking cessation advice as part of their duty. Also with regards to other sectors, occupational health nurses affirmed that their role is essential in smoking cessation interventions, but they did not believe they had the appropriate skill and training for this role (Ganz et al., 2015).

Our study also showed that smoking cessation counselling is significantly related only to nurses’ belief. Thus, it is essential to improve the nurses’ skills, knowledge and training, especially during pre-graduate training. It has been demonstrated that students preparing for a career in healthcare believed that healthcare professionals are role models for their patients and the community in general (Matranga et al., 2020). In the current literature, the evidence about nurses’ knowledge is conflicting. Lang, Waterworth & O’Brien (2018) found that 73.3% of their sample had a higher rate of nurses who received the appropriate education on smoking cessation advice. Conversely, the other authors (Mak, Loke & Wong, 2018) showed that half of the participants had not received training for smoking cessation interventions, and concluded that lack of education is not crucial for nurses. A number of studies consider the absence of education for nurses as a barrier to smoking cessation counselling, as the lack of basic knowledge seems to affect nurses’ awareness, and their ability to intervene with patients who smoke (Lancaster et al., 2000; Lang, Waterworth & O’Brien, 2018; McCarty et al., 2001; Sarna et al., 2000; Scanlon, Clark & McGuiness, 2008). Moreover, the efficacy of training courses to motivate nurses and make them proactive in improving their knowledge about smoking cessation counselling is acknowledged. For this reason, it is strongly recommended that nurses’ training includes time and incentive to undertake such activities (Malone et al., 2017).

Study limitations are inherent the cross-sectional study design. The cross-sectional survey does not allow drawing definitive conclusions about the attitude to engaging in smoking cessation counselling, but it can only suggest research hypotheses about the educational needs of tertiary care nursing staff. Other research is needed to investigate attitude, belief, and knowledge regarding smoking cessation counselling among primary and secondary care nurses.

Incorporation of smoking cessation interventions in nurses’ education could improve the nursing staff’s attitude, belief, and knowledge regarding smoking cessation counselling, which would lead to the inclusion of tobacco prevention and cessation as an integral part of patient care.

## Conclusions

One of the most effective smoking cessation strategies involves nurses, but they often lack of adequate expertise. Italian studies about smoking cessation knowledge of nurses are limited. Our paper achieved three important results on this topic: nurses of Cardiology, Cardiac Intensive Care and Surgical Oncology Units have considerable knowledge regarding the association between some chronic diseases and smoking; attitude towards smoking cessation counselling was significantly related to nurses’ belief about counselling; a sample of Italian nurses considered explaining the health benefits deriving from smoking cessation to be time consuming task. As a result, we can conclude that including this activity as part of the nurses’ responsibility can be a successful strategy to help inpatients quit smoking. In the next future, our team will focus on nurses belonging to lower care intensity wards, as we expect they have higher need for education on tobacco cessation techniques.

## Supplemental Information

10.7717/peerj.12213/supp-1 Supplemental Information 1 Raw data. Answers to the questionnaire from 77 nurses.

10.7717/peerj.12213/supp-2 Supplemental Information 2 STROBE Statement.

10.7717/peerj.12213/supp-3 Supplemental Information 3 Italian questionnaire.

10.7717/peerj.12213/supp-4 Supplemental Information 4 English questionnaire.

## Additional Information and Declarations

### Data Availability

The following information was supplied regarding data availability: The raw data are available in the Supplemental File.
