# Supplementary material for: Knowledge and attitudes towards smoking cessation counselling: an Italian cross-sectional survey on tertiary care nursing staff
Source: PeerJ. 2021 Oct 15;9:e12213. doi: 10.7717/peerj.12213 (PMC8522640; doi:10.7717/peerj.12213)
Supplement: Supplemental Information 3 [file peerj-09-12213-s003.pdf]

DATI ANAGRAFICI

Sesso: M ☐ F ☐

Età:

minore di 30 anni ☐

compresa tra 30 e 40 anni ☐

maggiore di 40 anni ☐

Titolo di studio

Scuola regionale per Infermieri ☐

Diploma universitario ☐

Laurea Triennale in Infermieristica ☐

Laurea Magistrale in Scienze Infermieristiche ed Ostetriche ☐

Unità Operativa di appartenenza

Cardiologia AOU Policlinico Paolo Giaccone (Palermo) ☐

Terapia Intensiva Cardiologica AOU Policlinico Paolo Giaccone (Palermo) ☐

Cardiologia ARNAS Ospedale Civico (Palermo) ☐

Terapia Intensiva Cardiologica ARNAS Ospedale Civico (Palermo) ☐

Chirurgia Oncologica ARNAS Ospedale Civico (Palermo) ☐

Chirurgia generale ad Indirizzo Oncologico AOU Policlinico Paolo Giaccone (Palermo) ☐

1. Segna con una crocetta le malattie e gli stati patologici per i quali pensi che il fumo di sigaretta sia un importante fattore causale:

Neoplasie maligne

☐ Cancro alla bocca

☐ Cancro alla laringe

☐ Cancro ai polmoni

☐ Cancro all'esofago

☐ Cancro all'uretere

☐ Cancro alla vescica

☐ Cancro all'utero

☐ Cancro ai reni

☐ Leucemia

☐ Cancro allo stomaco

☐ Cancro al pancreas

Malattie e disturbi delle vie respiratorie

☐ Malattie croniche polmonari ostruttive (BPCO)

☐ Polmonite

☐ Bronchite acuta e cronica

☐ Asma

Malattie cardiache e vascolari

☐ Malattie cardiache (infarto)

☐ Malattie cerebrovascolari (ictus)

☐ Aneurisma dell'aorta addominale

☐ Arteriosclerosi

Altre Malattie

☐ Sterilità

☐ Impotenza

☐ Complicazioni in gravidanza

☐ Cataratta

☐ Ulcera gastrica e duodenale

3

2. Sei un fumatore? (segna con una crocetta la risposta scelta)

SI ☐ NO ☐ EX-FUMATORE ☐

Per favore, se usi altri prodotti contenenti tabacco (es. pipa, sigaro), indicalo nello spazio sottostante:

____

____

3. Se sei un fumatore rispondi alle seguenti domande:

- Da quanto tempo fumi? _________________

- Quante sigarette fumi al giorno? _________________

Se usi altri tipi di prodotti contenenti tabacco, rispondi alle seguenti domande:

- Da quanto tempo li usi? _____________________

- Quante volte al giorno usi questi prodotti? ___________________

4. Ritieni di aver ricevuto una formazione (Corso di Laurea, Corsi di Perfezionamento, Master, ECM) adeguata sui danni alla salute indotti dal fumo di sigaretta?

(Segna con una crocetta la risposta scelta)

☐	☐	☐	☐	☐
**1=per niente**	**2=poco**	**3=abbastanza**	**4=molto**	**5=del tutto**

4

5. Quanto sei d'accordo con le seguenti affermazioni?
(Per favore, segna con una crocetta il tuo grado di accordo con ogni affermazione):

a) Il reparto degenti è il luogo ideale per fornire informazione sui danni alla salute indotti dal fumo di sigaretta

☐	☐	☐	☐	☐
**1=per niente**	**2=poco**	**3=abbastanza**	**4=molto**	**5=del tutto**

b) L'infermiere deve sempre chiedere al paziente fumatore se ha intenzione di smettere di fumare

☐	☐	☐	☐	☐
**1=per niente**	**2=poco**	**3=abbastanza**	**4=molto**	**5=del tutto**

c) Se il paziente manifesta l'intenzione di smettere di fumare, l'infermiere deve fissare una data precisa di inizio della cessazione

☐	☐	☐	☐	☐
**1=per niente**	**2=poco**	**3=abbastanza**	**4=molto**	**5=del tutto**

d) L'infermiere deve spiegare ai pazienti fumatori gli effetti nocivi del fumo sulla salute

☐	☐	☐	☐	☐
**1=per niente**	**2=poco**	**3=abbastanza**	**4=molto**	**5=del tutto**

e) L'infermiere deve spiegare ai pazienti fumatori i benefici per la salute che derivano dallo smettere di fumare

☐	☐	☐	☐	☐
**1=per niente**	**2=poco**	**3=abbastanza**	**4=molto**	**5=del tutto**

5

6. Quanto sei d'accordo con le seguenti affermazioni?
(Per favore, segna con una crocetta il tuo grado di accordo con ogni affermazione):

a) L'infermiere deve registrare sulla cartella clinica (anche in formato elettronico) la condizione di fumatore di ciascun paziente

☐ **1=per niente** ☐ **2=poco** ☐ **3=abbastanza** ☐ **4=molto** ☐ **5=del tutto**

7. Quanto sei d'accordo con le seguenti affermazioni?
(Per favore, segna con una crocetta il tuo grado di accordo con ogni affermazione):

a) L'infermiere deve mettere in atto interventi di counselling anti-fumo nell'ambito della propria attività clinica

☐ **1=per niente** ☐ **2=poco** ☐ **3=abbastanza** ☐ **4=molto** ☐ **5=del tutto**

b) Spiegare ai fumatori i benefici alla salute che derivano dallo smettere di fumare è un'attività difficilmente praticabile perché toglie tempo all'assistenza infermieristica

☐ **1=per niente** ☐ **2=poco** ☐ **3=abbastanza** ☐ **4=molto** ☐ **5=del tutto**

c) L'infermiere, nella maggior parte dei casi, possiede limitate conoscenze sugli effetti negativi del fumo sulla salute

☐ **1=per niente** ☐ **2=poco** ☐ **3=abbastanza** ☐ **4=molto** ☐ **5=del tutto**

d) L'infermiere non è in grado di aiutare un paziente fumatore a smettere di fumare

☐ **1=per niente** ☐ **2=poco** ☐ **3=abbastanza** ☐ **4=molto** ☐ **5=del tutto**

8. Quanto sei d'accordo con le seguenti affermazioni?

(Per favore, segna con una crocetta il tuo grado di accordo con ogni affermazione):

a) L'infermiere che consiglia ai pazienti fumatori di smettere di fumare li mette in imbarazzo

☐	☐	☐	☐	☐
**1=per niente**	**2=poco**	**3=abbastanza**	**4=molto**	**5=del tutto**

b) Se l'infermiere consiglia al paziente fumatore di smettere di fumare, il paziente lo considera un moralista

☐	☐	☐	☐	☐
**1=per niente**	**2=poco**	**3=abbastanza**	**4=molto**	**5=del tutto**

c) I pazienti fumatori stimano e apprezzano l'infermiere che consiglia loro di mettere di fumare

☐	☐	☐	☐	☐
**1=per niente**	**2=poco**	**3=abbastanza**	**4=molto**	**5=del tutto**

d) L'infermiere che consiglia ai pazienti di smettere di fumare indurrà molti fumatori a smettere di fumare

☐	☐	☐	☐	☐
**1=per niente**	**2=poco**	**3=abbastanza**	**4=molto**	**5=del tutto**

e) L'infermiere che consiglia ai pazienti di smettere di fumare mostra a medici e pazienti un'immagine migliore di sé

☐	☐	☐	☐	☐
**1=per niente**	**2=poco**	**3=abbastanza**	**4=molto**	**5=del tutto**

f) L'infermiere che consiglia ai pazienti di smettere di fumare contribuirà a ridurre l'incidenza delle malattie cardiovascolari e delle altre malattie correlate al fumo tra i fumatori

☐	☐	☐	☐	☐
**1=per niente**	**2=poco**	**3=abbastanza**	**4=molto**	**5=del tutto**

7
