# Supplementary material for: Knowledge and attitudes towards smoking cessation counselling: an Italian cross-sectional survey on tertiary care nursing staff
Source: PeerJ. 2021 Oct 15;9:e12213. doi: 10.7717/peerj.12213 (PMC8522640; doi:10.7717/peerj.12213)
Supplement: Supplemental Information 4 [file peerj-09-12213-s004.pdf]

English translation

SURVEY ON KNOWLEDGE AND ATTITUDE TOWARDS SMOKING CESSATION COUNSELLING IN THE DEPARTMENTS OF CARDIOLOGY, CARDIAC INTENSIVE CARE UNIT AND ONCOLOGY SURGERY OF THE AOUP AND CIVIC HOSPITAL OF PALERMO

We ask your cooperation in filling out the following questionnaire anonymously. The data will be used to assess the attitude and knowledge of nursing staff with regard to smoking cessation counselling. Counselling is a process that, through dialogue and interaction, helps people to solve and manage problems (in our case tobacco addiction) and to make decisions.

Personal data

Gender

□ Male □ Female

Age

□ < 30 □ Between 30-40 □ > 40

Education

□ Regional nursing school □ Nursing diploma □ Degree □ Master's degree

Membership hospital ward

□ Cardiology at AOUP "Paolo Giaccone" □ Cardiology at ARNAS Civico

□ Cardiac ICU at AOUP "Paolo Giaccone" □ Cardiac ICU at ARNAS Civico

□ Oncology at AOUP "Paolo Giaccone" □ Oncology at ARNAS Civico

Q1 Check the diseases for which you think cigarette smoking is an important causative factor:

Malignant neoplasms:

□ Mouth cancer □ Cancer of the oesophagus □ Laryngeal cancer □ Lung cancer

□ Ureter cancer □ Bladder cancer □ Cervical cancer □ Kidney cancer □Leukaemia □ Stomach cancer

□ Pancreatic cancer

Respiratory diseases and disorders:

□ Chronic Obstructive Pulmonary Diseases □ Pneumonia □ Acute and chronic bronchitis □ Asthma

Heart and vascular diseases:

□ Heart disease (heart attack) □ Cerebrovascular disease (stroke)

□ Abdominal aortic aneurysm □ Arteriosclerosis

Other diseases:

□ Infertility □ Impotence □ Complications in pregnancy

□ Cataract □ Gastric and duodenal ulcer

Q2 Are you smoker?

□ Yes □ Not □ Ex-smoker

Please, if you use other products containing tobacco (e.g., pipe, cigar), indicate this in the space below:

____

If you currently smoke:

Q3.1 How long have you been smoking? ____________________________

Q3 .2 How many cigarettes do you smoke a day? ____________________________

If you use other tobacco products, answer the following questions:

Q3.3 How long have you been using them? ____________________________

Q3.4 How many times a day do you use these products? ______________________

Q4. Do you believe you have received adequate training (Degree course, postgraduate courses, master, CME - Continuing Medical Education) on the health damage caused by cigarette smoking?

□ Absolutely Not
□ Not
□ Quite
□ Yes
□ Absolutely Yes

Q5a. The inpatient ward is the ideal place to provide information on the health damage caused by cigarette smoking

□ Absolutely Not
□ Not
□ Quite
□ Yes
□ Absolutely Yes

Q5b. The nurse must always ask the smoker patient weather she/he wants to quit smoking

□ Absolutely Not
□ Not
□ Quite
□ Yes
□ Absolutely Yes

Q5c. If the patient decides to quit smoking, the nurse must set a precise start date of smoking cessation
□ Absolutely Not
□ Not
□ Quite
□ Yes
□ Absolutely Yes

Q5d. The nurse must always explain to the smoker patient the harmful effects of smoking on health
□ Absolutely Not
□ Not
□ Quite
□ Yes
□ Absolutely Yes

Q5e. The nurse should explain the health benefits of quitting smoking to smoking patients
□ Absolutely Not
□ Not
□ Quite
□ Yes
□ Absolutely Yes

Q6. The nurse must register the smoking status of each patient on the medical record (also in electronic format)
□ Absolutely Not
□ Not
□ Quite
□ Yes
□ Absolutely Yes

Q7a. The nurse must do counselling interventions to smoking cessation in the context of clinical activity
□ Absolutely Not
□ Not
□ Quite
□ Yes
□ Absolutely YesQ7b. It is difficult to explain the health benefits deriving from smoking cessation because it takes time off nursing care
□ Absolutely Not
□ Not
□ Quite
□ Yes
□ Absolutely Yes

Q7c. In general, the nurse has limited knowledge of the negative effects of smoking on health

□ Absolutely Not
□ Not
□ Quite
□ Yes
□ Absolutely Yes

Q7d. The nurse is not able to help the smoker patient to stop smoking
□ Absolutely Not
□ Not
□ Quite
□ Yes
□ Absolutely Yes

Q8a. The nurse who advises smoker patients to quit smoking makes them uncomfortable
□ Absolutely Not
□ Not
□ Quite
□ Yes
□ Absolutely Yes

Q8b. If the nurse advises the smoker patient to stop smoking, the patient considers him a moralist

□ Absolutely Not
□ Not
□ Quite
□ Yes
□ Absolutely Yes

Q8c. Patients appreciate the nurse who advises them to stop smoking

□ Absolutely Not
□ Not
□ Quite
□ Yes
□ Absolutely Yes

Q8d. The nurse who advises patients to stop smoking will cause many smokers to quit smoking

□ Absolutely Not
□ Not

□ Quite
□ Yes
□ Absolutely Yes

Q8e. The nurse who advises patients to stop smoking gives a good picture of him/herself

□ Absolutely Not
□ Not
□ Quite
□ Yes
□ Absolutely Yes

Q8f. The nurse who advises patients to stop smoking will help reduce the incidence of cardiovascular disease and other smoking-related diseases among smokers

□ Absolutely Not
□ Not
□ Quite
□ Yes
□ Absolutely Yes
